# Epigenetic Changes and Functions in Pneumoconiosis

**DOI:** 10.1155/2022/2523066

**Published:** 2022-01-20

**Authors:** Yiping Li, Zhiwei Cheng, Hui Fan, Changfu Hao, Wu Yao

**Affiliations:** ^1^Department of Occupational and Environmental Health, College of Public Health, Zhengzhou University, No. 100 Science Avenue, Zhengzhou City, Henan Province, China; ^2^Department of Case Management, The Third Affiliated Hospital of Zhengzhou University, China; ^3^Ultrasonography Department, The Third Affiliated Hospital of Zhengzhou University, China; ^4^Department of Child and Adolecence Health, School of Public Health, Zhengzhou University, Henan, 450001, China

## Abstract

Pneumoconiosis is one of the most common occupational diseases in the world, and specific treatment methods of pneumoconiosis are lacking at present, so it carries great social and economic burdens. Pneumoconiosis, coronavirus disease 2019, and idiopathic pulmonary fibrosis all have similar typical pathological changes—pulmonary fibrosis. Pulmonary fibrosis is a chronic lung disease characterized by excessive deposition of the extracellular matrix and remodeling of the lung tissue structure. Clarifying the pathogenesis of pneumoconiosis plays an important guiding role in its treatment. The occurrence and development of pneumoconiosis are accompanied by epigenetic factors (e.g., DNA methylation and noncoding RNA) changes, which in turn can promote or inhibit the process of pneumoconiosis. Here, we summarize epigenetic changes and functions in the several kinds of evidence classification (epidemiological investigation, *in vivo*, and *in vitro* experiments) and main types of cells (macrophages, fibroblasts, and alveolar epithelial cells) to provide some clues for finding specific therapeutic targets for pneumoconiosis and even for pulmonary fibrosis.

## 1. Introduction

Pulmonary fibrosis is a chronic lung disease with myriad causes that is characterized by excessive deposition of the extracellular matrix (ECM) and remodeling of lung tissue structure. Pneumoconiosis, coronavirus disease 2019 (COVID-19), interstitial lung disease associated with systemic sclerosis (SSC-ILD), and idiopathic pulmonary fibrosis (IPF) all have such typical pathological changes. Currently, no specific treatment for pulmonary fibrosis exists. If pneumoconiosis occurs, pulmonary fibrosis is almost irreversible, even if there is no longer exposure to dust, and it can develop progressively. Pneumoconiosis is prevalent worldwide, especially in low-income and middle-income countries, and has a higher incidence among gold, iron, and tin mining workers. According to the nature of the mineral dust that causes pneumoconiosis, the disease can be divided into the following categories: (1) silicosis caused by dust containing mainly free silica, which is the most common type of pneumoconiosis; (2) silicate pneumoconiosis caused by silicate-based dust, including asbestos pneumoconiosis, talc pneumoconiosis, cement pneumoconiosis, mica pneumoconiosis, and potter's pneumoconiosis; (3) pneumoconiosis caused by coal dust and carbon-based dust, including coal worker's pneumoconiosis, graphite pneumoconiosis, and carbon black pneumoconiosis; and (4) metal pneumoconiosis caused by metal dust, including aluminum pneumoconiosis, welder's pneumoconiosis, and caster's pneumoconiosis. China is the country most seriously affected by pneumoconiosis in the world, with the highest numbers of dust exposure, new cases of pneumoconiosis, and current cases of the disease. Although the global incidence of pneumoconiosis has decreased in recent years, the number of people living with pneumoconiosis remains high. In China alone, the cumulative number of patients with pneumoconiosis exceeded 6 million as of 2013. In addition, pneumoconiosis cannot be effectively treated, so the economic and health burdens of pneumoconiosis on the country, society, family, and individuals remain severe. The cost of pneumoconiosis for patients in China is approximately 19,000 RMB/person-year (medical expenses) plus 46,000 RMB/person-year (indirect management expenses). The average life expectancy of pneumoconiosis after diagnosis is 32 years. Given the current economic market, the fiscal burden of pneumoconiosis is approximately RMB 2.075 million/person [1–4].

Epigenetics refers to changes in gene expression and function without changing the DNA sequence, and this phenotypic change can be inherited. It plays a key role in many cellular processes, such as gene expression regulation, cell proliferation and differentiation, and chromosome inactivation [5]. Epigenetic regulation can be divided into gene-selective transcription regulation, gene posttranscription regulation, and protein posttranslational regulation. Currently, epigenetic studies have focused on selective and posttranscriptional regulation of genes, including DNA methylation, noncoding RNA (ncRNA) regulation, histone modification, and chromatin remodeling [5]. In pulmonary fibrosis, the epigenetic changes are obvious. These changes also play an important role in the occurrence and development of pneumoconiosis, mainly through changes in DNA methylation and ncRNA regulation.

## 2. DNA Methylation

DNA methylation is the main epigenetic mechanism of gene selective transcription regulation. DNA methylation refers to the process in which DNA methyltransferases (DNMTs) transfer the methyl group of S-adenosylmethionine to a specific base on DNA. In mammals, methylation occurs on the fifth carbon atom of the cytosine (C) of the 5'—C—phosphate—G—3'(CpG) dinucleotide, which is separated from guanine (G) by a phosphate to form 5-methylcytosine (5mC) [6, 7].

The distribution of CpG dinucleotides is not uniform on DNA. The CpG in some areas presents as an aggregated state, usually 1-2 kb, called a CpG island. Approximately 70% of gene promoter regions in mammals contain CpG islands, and most of them are in an unmethylated state. The methylation degree of CpG islands is closely related to gene transcription. Approximately 70-80% of CpG sites not in islands are in a methylation state [8]. Other locations on DNA that are associated with CpG islands include CpG shores, which are located within 2 kb upstream and downstream of CpG islands; CpG shelves, which are located within 2 kb upstream and downstream of CpG shores; and open sea CpGs, which are located in areas other than the island, shores, and shelves [9, 10]. Generally, the methylation of CpG islands in the promoter region is related to gene expression inhibition, and DNA methylation in the gene body is related to gene activation [6, 11].

### 2.1. DNA Methylation Patterns

Maintenance methylation refers to the same methylation modification on the nascent DNA strand as that on the parent strand during cell division. De novo methylation refers to the methylation of unmethylated CpG sites.

### 2.2. DNA Methylation–Related Enzymes

DNA methylation or demethylation is mainly carried out by numerous enzymes. The role of DNA methylation in mammals is mainly mediated by DNMTs and methyl-CpG-binding proteins (MBPs) [12–17].

#### 2.2.1. DNMTs

DNMTs include DNMT1, DNMT2, and DNMT3. DNMT1, also known as maintenance methyltransferase, is highly expressed in various mammalian tissues. Methylation catalyzed by DNMT1 can be inherited. DNMT2 is also widely distributed in human and murine tissues, but its role is unclear. DNMT3 subtypes include DNMT3a, DNMT3b, and DNMT3L. DNMT3a and DNMT3b are also known as de novo DNMTs. DNMT3a is widely distributed; DNMT3b is mainly distributed in the thyroid, testis, and bone marrow. DNMT3L assists DNMT3a and DNMT3b, because it does not have catalytic activity alone [18].

#### 2.2.2. MBPs

DNA methylation in the promoter region can directly interfere with the binding of transcription factors to cis-acting elements, leading to a decrease in gene transcription. In addition, DNMT1 [19] and DNMT3a [20] can change the chromatin structure and inhibit gene transcription by binding to the histone methyltransferase SUV39H1. Methylation mediated by MBPs is the most common form of gene expression silencing caused by DNA methylation. MBPs include methyl-CpG binding domain protein (MBD); ubiquitin-like, containing PHD and RING finger domain protein (UHRF); and zinc finger protein. Five main types of MBD proteins exist in mammals: methyl-CpG binding protein 2(MeCP2), MBD1, MBD2, MBD3, and MBD4. They all have conserved CpG-binding domains, and the first three have transcriptional inhibitory domains that can recruit transcriptional inhibitors. The main function of the UHRF protein is not to inhibit gene transcription but to maintain DNA methylation during DNA replication that occurs in cell division and proliferation. Zinc finger proteins, including KAISO, ZBTB4, and ZBTB38, bind methylated DNA through zinc finger domains, thereby inhibiting gene transcription. Among them, KAISO preferentially binds to two consecutive CpG sites [18].

### 2.3. DNA Demethylation

DNA methylation is a reversible process. DNA demethylation includes (1) passive DNA demethylation, in which maintenance methyltransferases are inhibited or dysfunctional, and the level of DNA methylation gradually decreases with cell division and proliferation; and (2) active DNA demethylation, which is regulated mainly by deaminase activation-induced deaminase (AID)/apolipoprotein B mRNA-editing catalytic polypeptide-like (APOBEC) and translocation t(10; 11) [18, 21–24]. Tet catalyzes the hydroxylation of 5mC to produce 5-hydroxymethylcytosine. In mammals, genomes undergo two large-scale active DNA demethylations: one during early embryonic development from zygote to implantation and one during gamete formation. Other small-scale active demethylation occurs at specific sites locally in somatic cells. DNA methylation and DNA demethylation balance each other, and the disorder of either will lead to phenotypic changes and potential disease development.

### 2.4. DNA Methylation and Pneumoconiosis

**Figure 1:**
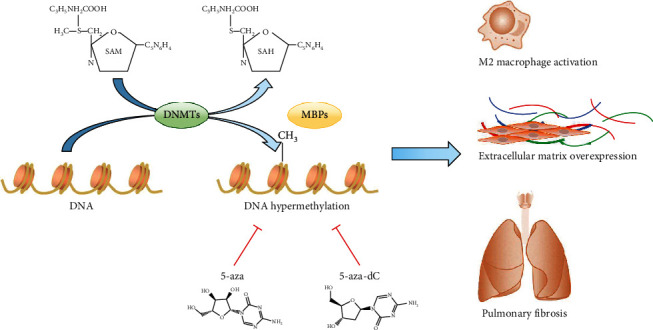
The main DNA methylation changes involved in pneumoconiosis.

DNA methylation has been extensively studied in pulmonary fibrosis diseases, such as COVID-19, SSC-ILD, and IPF. DNA methylation is also involved in the pathogenesis of fibrosis in multiple organs and systems. For example, DNA methylation can regulate myofibroblast differentiation and apoptosis resistance [25, 26], leading to significant changes in morbidity and mortality. However, the study of DNA methylation in pneumoconiosis is not in-depth, and most studies simply observe the changes in DNA methylation. The main DNA methylation changes involved in pneumoconiosis are shown in Figure 1.

The study by de Souza et al. [27] analyzed the relationship between coal miners' telomere lengths (TLs) and the percentages of DNA methylation in the entire genome showed that occupational exposure to coal and combustion products positively correlated with TL and DNA methylation. Additional research is needed to determine whether these changes are related to the outcome of the induced disease and whether these events may be determinants of cancer risk. Qian and Zhang et al. [28–30] used an Illumina Human Methylated 450K Bead Chip Array to detect methylation changes in formalin-fixed, paraffin-embedded (FFPE) lung specimens of human silicosis. Compared with data on normal lung methylation from the Gene Expression Omnibus Database (GEO), the specimens contained 86,770 CpG sites in the early stage of silicosis and 79,660 CpG sites in the late stage; differences in methylation were significant [28–30]. Kyoto Encyclopedia of Genes and Genomes analysis showed that the methylation state of the MAPK signaling pathway changed most obviously. Among the changes noted, the hypermethylation of the phosphatase and tensin homolog deleted on the chromosome 10 (PTEN) promoter was considered potentially related to the reduction of its protein [28–30]. Umemura et al. [31] found that detection of aberrant promoter hypermethylation of tumor suppressor genes in DNA from serum of patients with silicosis may help identify early lung cancer.

Thy-1 DNA methylation has been extensively studied in other pulmonary fibrosis diseases, and an increase in its methylation plays an important role in pneumoconiosis [32, 33]. In a case-control study of pneumoconiosis by Ci, Thy-1 DNA hypermethylation occurred in the peripheral blood of patients with pneumoconiosis through the effects of DNMT3B and MeCP2 [34]. Conghui et al. [35] infected THP-1 macrophages with coal dust or silica dust, collected the macrophage supernatant, and then stimulated human embryonic lung fibroblast MRC-5 cells with the collected supernatant. The study ascertained that, in the process of MRC-5 cells fibrosis, Thy-1 DNA methylation was increased and Thy-1 expression was decreased. In a coal worker's pneumoconiosis (CWP) model of specific-pathogen-free (SPF) male Sprague-Dawley (SD) rats and rat alveolar type II cells (RLE-6TN) established by Zhang, the protein expressions of DNMT1, DNMT3a, DNMT3b, MBD2, and MeCP2 changed compared with the control group. Dust exposure can induce hypermethylation of Thy-1 DNA in RLE-6TN cells and in rats. Pretreatment of RLE-6TN cells and rats with the DNMT inhibitor 5-aza-2′-deoxycytidine (5-aza-dC) can reduce the fibrosis [36]. In a trans-well culture model of rat primary alveolar macrophages and rat primary lung fibroblasts by Hou and Li et al. [37–39], DNA methylation levels of a large number of genes were altered during the SiO_2_-induced fibroblast to myofibroblast progression. These changes mainly occur in the metabolic process, biological regulation, and biological adhesion pathways. In an experiment by Wang et al. [15, 40], the expressions of DNMT1, DNMT3a, and MBD2 in primary lung fibroblasts of rats were reduced in a dose-dependent manner under the action of macrophages stained with micron-sized SiO_2_ particles, and the genome-methylation level was also reduced in a dose-dependent manner. DNA methylation has not been widely used in pneumoconiosis research and clinical practice, but DNMTs inhibitors, especially 5-azacytidine (5-aza-C) and 5-aza-dC (which are common in cancer treatment), have been used for pneumoconiosis treatment [41]. MeCP2 is essential for myofibroblast differentiation and lung fibrosis. Xiang et al. [42] found that MeCP2 was correlated with *ACTA2* expression in primary IPF fibroblasts through a chip experiment. MeCP2 plays a key role in the upregulation of *ACTA2* by transforming growth factor *β* (TGF-*β*) in lung fibroblasts, and 5-azaC can partially block the expression of *ACTA2* induced by TGF-*β* [42]. Although DNMTs inhibitors have been widely used for treatment, their disadvantages are obvious. Currently available DNMT inhibitors lack specificity for the gene of interest; however, site-specific treatments based on genome editing technology or regulation by ncRNAs are in development, which may address some challenges associated with these treatments [41, 43, 44]. In one study, inhibition of DNMT3 or telomere reverse transcriptase (TERT) with siRNA reduced the aging and fibrosis process of A549 cells that was induced by TGF-*β* [45]. Jianghui and Wang et al. [46, 47] ascertained that MBD2 was highly expressed in M2 macrophages in the bronchoalveolar lavage fluid from lungs affected by different types of pulmonary fibrosis, including COVID-19, SSC-ILD, and IPF, and in murine models of pulmonary fibrosis that were established using bleomycin (BLM). MBD2-knockout mice showed reduced M2 macrophages and reduced lung injury and fibrosis [46, 47]. Other treatments for IPF, such as the bromodomain containing 4 (BRD4) inhibitor JQ1, the type-2 histone deacetylase 2 (HDAC) inhibitor spiruchostatin A, and suberoylanilide hydroxamic acid, may also be useful for treatment of pneumoconiosis [48–50].

Methylation changes do exist in pneumoconiosis, but a full assessment of the specific changes and their effects requires large-scale epidemiological studies and *in vivo* and *in vitro* experiments. Little research on DNA methylation–related treatments for pneumoconiosis exists, but research in other fibrotic diseases, especially IPF, can be used as a reference.

## 3. NcRNA

**Figure 2:**
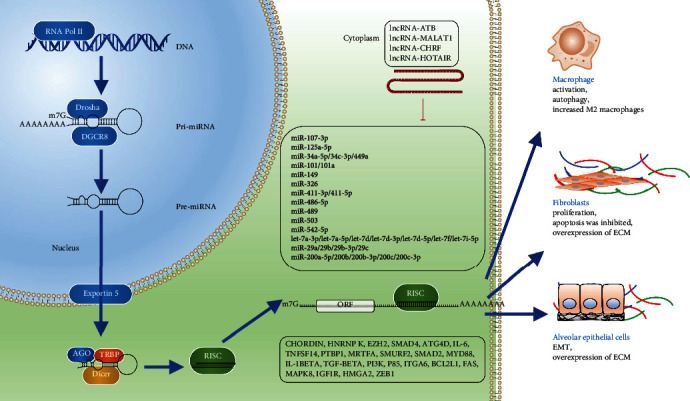
The main noncoding RNA (ncRNA) changes involved in pneumoconiosis.

NcRNA refers to RNA that cannot be translated into protein. A small part of ncRNA, called housekeeping ncRNA or infrastructural ncRNA (e.g., rRNA and tRNA), is expressed constitutively. Expression of most ncRNAs is induced under certain conditions; regulatory ncRNAs can manipulate mRNA expression. Regulatory ncRNAs can be divided into long ncRNA (lncRNA) and short-strand ncRNA (<50 nt) according to their molecular sizes, and short-strand ncRNAs can be divided into microRNA (miRNA), small interfering RNA (siRNA), and piwi-interacting RNA (piRNA) [18]. Circular RNA (circRNA) is a recently discovered ncRNA, and its main feature is that the molecule is in a closed loop. In pulmonary fibrosis diseases, miRNA is the most studied ncRNA, followed by lncRNA. Regulatory ncRNA plays a role in the regulation of gene-selective transcription and gene posttranscription regulation. The main ncRNA changes involved in pneumoconiosis are shown in Figure 2.

### 3.1. miRNA

#### 3.1.1. Production and Function of miRNA

The classic method of miRNA production involves corresponding independent genes for each miRNA that are transcribed by RNA polymerase II to produce primary precursors and become pri-miRNA. Pri-miRNA is processed in the nucleus by the Drosha-DGCR8 complex into pre-miRNA, which has a hairpin structure. Pre-miRNA is processed by Dicer in the cytoplasm into miRNA/miRNA^∗^ duplexes. Usually, miRNA^∗^ is degraded, and miRNA plays a biological role [18]. In addition, miRNA can also be produced from introns or lncRNA.

The expression of miRNA also regulated by many factors. As in mRNA, the gene promoter region of miRNA also contains CpG islands, TATA boxes, and transcription initiation elements, and the transcription of miRNA is also regulated by transcription factors, enhancers, and silencers [18, 51, 52]. The expression of miRNA is also regulated by epigenetic factors, such as DNA methylation, lncRNA, circRNA, and histone modification [22, 53–59]. Many miRNAs are also regulated in posttranscriptional processing; for example, miRNAs are spliced by argonaute (AGO) proteins [60], Drosha [61, 62], Dicer [63], silencing regulatory protein KRSP [64], and tristetraprolin (TTP) [64]. The stability of miRNA is another factor that affects its expression level. For example, the half-life of miRNA-29b in HeLa cells is approximately 4 h, whereas the half-life of miRNA-29a in the same cell line is longer than 12 h, which contributes to the different miRNA expression levels [18]. The stability of miRNA is regulated mainly by miRase, and some target mRNAs can also regulate the stability of miRNA via negative feedback. The biological functions of mature miRNAs occur mainly in the cytoplasm. miRNAs can indirectly regulate gene expression by regulating HDAC [65] and DNA methylase [66]. In addition, miRNA can form an miRNA-induced silencing complex (miRISC) [61], thus, promoting mRNA degradation and directly inhibiting mRNA translation into protein. Some pre-miRNAs in the nucleus can also competitively bind repressor proteins and facilitate mRNA translation into proteins [67, 68].

#### 3.1.2. miRNA and Pneumoconiosis

Many studies have explored miRNA because of the early discoveries about its relevance to various diseases throughout the body. miRNA plays important roles in the occurrence and development of pneumoconiosis, and these roles are summarized in Table 1. Pneumoconiosis involves a state of constant change, and miRNA has a certain space-time specificity; in addition, miRNA has multiple target genes. Finding key changes in this complex network is the focus of current research. Currently, miRNA studies in pneumoconiosis mainly focus on finding significantly changed miRNAs in human or animal models as indicators for a disease diagnosis or treatment; some studies explore the target genes regulated by miRNAs and their related pathways in animal or cell models to provide a theoretical basis for the treatment of diseases.

*(1) Case-Control Study*. Use of miRNA sequencing or microarrays in case-control studies can help identify miRNAs that have been explored and changed significantly. Using a TaqMan low-density core, Yitao [69] screened out the increased expression of miR-16, miR-21, miR-29a, miR-155, miR-200c, and miR-206—and the decreased expression of miR-204—in the serum of patients with pneumoconiosis enrolled in a case-control study. This study established a multiserum miRNA combined regression model, which is expected to be applied in the early screening and diagnosis of pneumoconiosis [69]. Huang et al. [80] performed miRNA detection on four patients with pneumoconiosis and on four matched healthy controls; 1,079 miRNAs with different changes were screened out, among which 406 were upregulated and 117 were downregulated. Gene Ontology (GO) analysis revealed that the changed miRNAs were mainly involved in the biological processes affected by hemophilic cell adhesion, anatomical structure development, and cell–cell adhesion via plasma membrane proteins [80]. Rong et al. [98] observed a decrease in the expression of miR-200c and miR-29c in the serum of dust-exposed workers with severely decreased lung function and noted an increase in ECM protein. Guo et al. [90] used miRNA microarray to determine that miR-222 was significantly downregulated in the serum samples of patients with CWP, whereas miR-671-3p was significantly upregulated.

The difference in genotype not only affects the susceptibility to diabetes and cancer but also seems to affect the susceptibility to pneumoconiosis. In a case-control study of 496 patients with CWP and 513 controls, Wang et al. [102] discovered that the CWP risk significantly increased when the miR-149 rs2292832 TT genotype was present.

*(2) In Vivo or In Vitro Experiments*. In addition to studies that macroscopically examine the role of miRNA in pneumoconiosis, many studies are focusing on pathogenesis of pneumoconiosis; the mainstream view is that respirable productive dust enters the lung and first contacts alveolar macrophages and alveolar epithelial cells. The entry and retention of the vast majority of exogenous irritants (dust, allergens, chemical poisons, etc.) in the lungs will first cause macrophage alveolitis. Macrophages phagocyte the dust, and some macrophages carry the dust into the interlobular septum, which backflows to lymph nodes, blood vessels, and nearby bronchus and other parts. Because the dust cannot be dissolved or discharged, the dust will stay in the lung tissue for a long time and form a dust cell granuloma. With the constant existence of dust and the continuous tissue damage and repair, chronic inflammation and excessive repair processes develop; thus, a large number of myofibroblasts and ECM deposits are produced and eventually lead to fibrosis [103–105].

*Macrophages.* In the process of pneumoconiosis lung fibrosis, macrophages play an extremely important initial role. Zhang et al. [73, 106] found through miRNA microarray that the expression of 14 miRNAs in the fibrotic lungs of rats with silicosis was significantly upregulated, whereas the expressions of the other 25 miRNAs were significantly downregulated. Among these 25, the expression level of miR-146a increased significantly, and the expression level of miR-181b decreased significantly [73, 106]. The researchers detected a negative correlation between miR-181b and tumor necrosis factor- (TNF-) *α* and between miR-146a and interleukin- (IL-) 1*β* by overexpression or inhibition of miRNA in SiO_2_-treated NR8383 cells [73, 106]. These altered miRNAs may affect the occurrence and development of silicosis by regulating the inflammatory response of macrophages [73, 106].

Macrophage autophagy is also a hot spot for miRNA changes in pneumoconiosis. Chen et al. [85–87] ascertained that the expression of miRNA-101 decreased in a rat model of silicosis and in NR8383 cells after exposure to silica dust. Transplantation of bone marrow mesenchymal stem cells (BMSCs) overexpressing miRNA-101 had a significantly better therapeutic effect on fibrosis in rats with silicosis compared with simple BMSC transplantation [85–87]. BMSC with miRNA overexpression can inhibit the excessive activation of autophagy in lung tissue after being infected with dust, reduce chronic inflammation, and delay pulmonary fibrosis in patients with silicosis [85–87]. *In vitro* experiments discovered that overexpressed miRNA-101 in BMSCs can inhibit autophagy related 4D cysteine peptidase (ATG4D) by regulating the endoplasmic reticulum stress of NR8383 cells and effectively inhibit the autophagy of macrophages caused by silica [85–87].

*Fibroblasts.* Fibroblasts are the main source of myofibroblasts, and the changes and effects of miRNA in pneumoconiosis-related fibroblasts have been studied the most. Through miRNA sequencing, Zhang et al. screened nine miRNAs in human serum exosomes from case-control studies on silicosis; in this study, the expression of let-7a-5p, let-7i-5p, miR-146b-5p, miR-151A-3p, miR-409-3p, and miR-423-3p decreased, but the expression of miR-96-5p, miR-122-5p, and miR-125a-5p increased [68, 74, 75]. Exosomal miRNA plays an important regulatory role in the pathogenesis of pneumoconiosis, mainly via autophagy, apoptosis, the PI3K-Akt signaling pathway, the MAP signaling pathway, and other methods [68, 74, 75]. Cell experiments have ascertained that the overexpression of let-7a-5p and let-7i-5p in human lung fibroblasts (IMR-90 cells) contributed to the reduction of fibrosis [68, 74, 75]. To identify target genes of the exosome let-7a-5p, researchers compared all potential target genes with the Malacards platform and identified four target genes: *BCL2L1*, *FAS*, *MAPK8*, and *IGF1R* [68, 74, 75]. They also detected that miR-125a directly inhibited the expression of *hnRNP K* and *EZH2* [68, 74, 75].

Ji et al. [70, 107] found by miRNA microarray screening that miR-21 was upregulated and that miR-455, miR-486-5p, and miR-3107 were downregulated in lung tissue from a murine model of silicosis. The expression of miR-486-5p was also downregulated in the serum and lung tissue of patients with silicosis and in the lung tissue of patients with IPF. Overexpression of miR-486-5p can alleviate lung fibrosis induced by silica and BLM in mice. *In vitro* TGF-*β* stimulation of NIH/3T3 showed that miR-486-5p could bind to SMAD2 and participate in the process of pulmonary fibrosis through the TGF-*β* signaling pathway.

In a study by Gao et al. [71, 79], 70 differentially expressed miRNAs were screened by high-throughput sequencing in the lung tissue of a rat model of silicosis; of these 70 miRNAs, 41 were upregulated and 29 were downregulated. Primary rat lung fibroblasts stimulated by TGF-*β in vitro* showed that miR-293-5p, miR-370-3p, miR-409a-5p, miR-411-3p, miR-555-3p, and miR-1194-3p were consistent with the previous sequencing results. Using miRNA mimics and inhibitors, researchers discovered that miR-370-3p, miR-411-3p, and miR-1193-3p had antifibrosis effects, whereas miR-293-5p had a profibrosis effect [71, 79]. They also found that miR-411-3p effectively reduced the progression of silicosis or fibroblast fibrosis by regulating the expression of myocardin-related transcription factor A (MRTFA) *in vivo* and *in vitro* [71, 79]. In addition, miR-411-3p inhibited the expression of SMAD ubiquitination regulatory factor 2 (SMURF2) and reduced the ubiquitination degradation of SMAD7 regulated by SMURF2, thereby blocking the TGF-*β*/SMAD signaling pathway and alleviating silicosis-related pulmonary fibrosis by stimulating the primary lung fibroblasts of rats with TGF-*β* [71, 79].

Yuan et al. [96] ascertained that the expression of miR-542-5p was downregulated in a murine model of silicosis in which NIH/3T3 cells were treated with TGF-*β*. Overexpression of miR-542-5p can alleviate fibrosis [96]. Integrin alpha 6 (ITGA6), a cell surface protein associated with fibroblast proliferation, was demonstrated to be a direct target of miR-542-5p [96]. Downregulation of ITGA6 significantly inhibited the phosphorylation of FAK/PI3K/AKT [96].

*Alveolar Epithelial Cells.* In addition to macrophages, alveolar epithelial cells are the first cells to directly contact dust. Alveolar type II epithelial cells are transformed into myofibroblasts through the epithelial mesenchymal transdifferentiation (EMT) process, and they are also one of the main sources of myofibroblasts in pneumoconiosis. Jiayu [72] discovered using miRNA microarray chips that miR-21-5p and miR-423-5p were upregulated and that miR-7d-3p, miR-26a-5p, miR-29b-3p, and miR-34c-3p were downregulated in lung tissues of rats with silicosis. These miRNAs are mainly involved in signal transduction, gene transcription, inflammation, apoptosis, and the cell cycle. Subsequently, these researchers verified the downregulation of miR-29b-3p and miR-34c-3p in A549 cells treated with silica and TGF-*β* [72]. In the serum of patients with silicosis, the expression of miR-21-5p increased with the progression of disease, whereas the expression of miR-29b-3p and miR-34c-3p decreased [72]. Using whole-genome sequencing, Shasha [88] found in a case-control study of CWP that the expression of miR-25 and miR-9 was upregulated in each stage of pneumoconiosis and that the expression of miR-20b, miR-222, and miR-149 was downregulated. These miRNAs are mainly involved in signal transduction, gene transcription, inflammation, cell proliferation, apoptosis, and the cell cycle [88]. These researchers also detected increases in the serum IL-6 in the case group as the disease progressed [88]. The downregulated expression of miR-149 and the increased level of IL-6 were also confirmed in a murine model of silicosis, in a silica stimulation of A549 cells, and in a human bronchial epithelial (HBE) cell model [88]. Overexpression of miR-149 can inhibit the expression of IL-6 in cell experiments [88]. Sun et al. [89] detected silica-treated A549 cells with microarray and ascertained the changes of miR-200c, miR-149, and miR-29b; then, they verified the downregulation of miR-29b expression in RLE-6TN cells. Through cell and animal experiments, the researchers found that overexpression of miR-29b attenuates or even reverses the EMT process induced by silica. Yu et al. [97, 108] discovered that THP-1 macrophages that phagocytose micron-sized silica particles can reduce the expression of let-7d increase the expression of HMGA2 and then promote the EMT process of A549 cells. Zhao and Qi et al. [81, 82] detected that the expression of miR-34a-5p decreased, and the expression of SMAD4 increased in murine models of silicosis and TGF-*β*–stimulated A549 cells. Additional cell experiments proved that miR-34a-5p may play a role in inhibiting EMT by inhibiting SMAD4 [81, 82].

*Combined Studies of Different Cells.* The research of Zhang et al. [76–78, 109] skillfully combined two key cells in pneumoconiosis, the initiating macrophages, and the effector cell fibroblasts. In a case-control study of silicosis, they ascertained that the miRNA profile in human serum exosomes was altered; then, they stimulated murine RAW264.7 macrophages with micron-sized silica particles and discovered that the macrophages secreted more exosomes and that the miRNA profile altered [76–78, 109]. After intersecting the miRNA that was differentially expressed in human serum and in the RAW264.7 exosome, the expressions of miR-30c-2-3p, miR-107-3p, miR-122-5p, miR-125a-5p, miR-126a-5p, and miR-335-5p were increased, and the expression of miR-27b-5p was decreased [76–78, 109]. Among them, miR-107-3p can inhibit the expression of chordin and downstream BMP-2, SMAD1/5/9 and ID-1, which are related to the activation of the TGF-*β* signaling pathway [76–78, 109]. miR-125a-5p can promote the proliferation and apoptosis of the murine NIH/3T3 fibroblasts and promote the occurrence of fibrosis [76–78, 109].

Han and Fan et al. [83, 84, 110] combined the two most important cell changes in pneumoconiosis-related lung fibrosis with the same miRNA. They found that, during the occurrence and development of silicosis, fibroblasts have a different autophagy pattern from macrophages. In a murine model of silicosis, the expression of miR-449a increased during the inflammatory phase but gradually decreased with the development of lung fibrosis, and the autophagy activity of lung tissue cells was also inhibited. The expressions of NOTCH1 and SNAIL were opposite the expression of miR-449 [83, 84, 110]. After overexpression of miR-449a, the course of EMT and the fibrosis in mice with silicosis were relieved [83, 84, 110]. This response was also verified in a fibrosis model of NIH/3T3 cells and human embryonic lung fibroblast MRC-5 cells stimulated by TGF-*β in vitro*, and the research demonstrated that miR-449a alleviated pulmonary fibrosis and promoted fibroblast autophagy by inhibiting its target, Bcl2 [83, 84, 110]. The researchers also stimulated HBE cells *in vitro* with silica and demonstrated that miR-449a inhibits NOTCH1, which in turn inhibits SNAIL and EMT [83, 84, 110].

Among the epigenetic changes of pneumoconiosis, miRNA is the most widely studied. A large number of miRNA microarrays and sequencing of human and animal peripheral blood and lung tissues provide biomarkers for disease screening, diagnosis, and prognosis. At the same time, many studies have explored the specific role of miRNA, mainly focusing on miRNA regulation of fibrosis-related target genes. Because of the temporal and spatial specificity of miRNA changes, the search for the key changes of miRNA has become the focus of this kind of research. However, the therapeutic potential of miRNA has not yet been tapped, and the antifibrosis effect remains explored mainly in cell and animal experiments. The treatment of liver fibrosis with miRNA has been widely applied in clinical practice, and clinical trials are exploring miRNA in the treatment of renal fibrosis, but few clinical studies have studied miRNA in the treatment of pulmonary fibrosis [52, 111]. Looking for miRNAs that can be used as targets can provide new ideas for the treatment of pneumoconiosis and even pulmonary fibrosis.

### 3.2. lncRNA

#### 3.2.1. Production and Function of lncRNA

The coding sequence of lncRNA is widely distributed in the genome. Most lncRNAs are produced from the transcription of protein-coding intergenic sequences. A small part of RNA produced by reverse transcription of protein-coding genes or secondary promoters in pseudogenes is called the natural antisense transcript (NAT). These NATs could play a direct role as lncRNAs, or they could bind to the mRNA generated by the transcription of the parent encoding genes to form double-stranded RNA [112].

Some lncRNA expressions are regulated by transcription factors or epigenetics [113–115], and some lncRNA expressions are directly induced by ionizing radiation or DNA damage [116–118]. Stability of the lncRNA is mainly regulated by RNA-binding proteins and miRNAs [18].

There are many types of lncRNA, and their functions are also diverse. The function of lncRNA is related to its location. lncRNAs in the nucleus are mainly involved in the regulation of mRNA and protein synthesis. For example, the combination of lncRNA and the chromatin remodeling complex promotes the formation of a dense chromatin structure, thereby generating gene-silencing regions [119]. lncRNA can also regulate histone modification [120], promote DNA methylation modification [121], inhibit transcription factors [122], and inhibit RNA polymerase II [123] to regulate mRNA expression, or NATs can directly interfere with mRNA expression, posttranscriptional processing, and transportation [112, 124, 125]. lncRNAs located in the cytoplasm are mainly involved in the regulation and transportation of protein translation. lncRNAs can promote the translation of mRNA through the mechanism of competing endogenous RNAs (ceRNAs) [91–95, 99, 100], or inhibit the translation of mRNA [126], or directly promote the degradation of mRNA [127, 128]. Even the same lncRNA may have different functions when it is in different locations. For example, lncRNA-HOTAIR located in the nucleus regulates histone modification and chromatin remodeling [60, 65, 129, 130], whereas those located in the cytoplasm can bind miRNA through the ceRNA mechanism [66, 131–133].

#### 3.2.2. lncRNA and Pneumoconiosis

Like studies of miRNAs, studies of lncRNAs in pneumoconiosis are mainly divided into two topics: (1) lncRNAs that directly regulate target genes related to fibrosis; (2) lncRNAs that compete with miRNAs in binding target genes. The main lncRNA changes involved in pneumoconiosis are summarized in Table 2.

*(1) Case-Control Study*. Use of an lncRNA microarray or of sequencing technology is conducive to the detection of typical differences in disease variation. Sai et al. [135] used an Agilent Rat lncRNA Array 8 × 60 K microarray to assess lung tissue of rats with silicosis and detected that silica exposure could alter the expression profile of 682 lncRNAs (of which 300 were upregulated and 382 were downregulated). The research predicted 73 pairs of ceRNAs [135]. The target genes involved in 13 pathways were mainly related to protein binding, cell shape, and exosomes [135].

The genotypes of lncRNAs also have a certain influence on the susceptibility to pneumoconiosis. In a case-control study of 703 patients with CWP and 705 control patients, Wu et al. [136] genotyped three potential polymorphisms (rs2067051, rs217727, and rs2839702) in lncRNA-H19. This study shows that the SNP rs2067051 of H19 is associated with a reduced risk of CWP in the Chinese population, providing a new genetic marker for screening and early intervention in high-risk populations of CWP [136].

*(2) In Vivo or In Vitro Experiments*. In addition to macroscopically studying the differences of lncRNA in case-control studies, many experiments have also penetrated into the pathogenesis of pneumoconiosis through *in vivo* and *in vitro* intervention experiments. The mechanism of lncRNA is mainly studied in two sources of myofibroblasts: fibroblasts and alveolar epithelial cells.

*Fibroblasts.* Cai et al. [134] sequenced the lncRNAs of rats with silicosis and ascertained that 306 lncRNAs were differentially expressed in the lungs of rats with silicosis, among which 224 were upregulated and 82 were downregulated. They subsequently verified that the expressions of LOC103691771, LOC102549714, and LOC102550137 were upregulated and that the expressions of LOC103693125 and LOC103692016 were downregulated in rats with silicosis and in TGF-*β*1–induced primary rat fibroblasts [134]. In addition, LOC103691771 promoted differentiation of myofibroblasts by regulating the TGF-*β*1–SMAD2/3 signaling pathway [134].

*Alveolar Epithelial Cells.* A case-control study by Ma et al. in CWP discovered that lncRNA-ATB was generally elevated in patients and was significantly, positively correlated with peripheral blood TGF-*β* in patients; thus, TGF-*β* may be a biomarker for CWP [101]. Liu established a TGF-*β*–induced EMT model using A549 cells and human bronchial epithelial (BEAS-2B) cells and found that the expression of lncRNA-ATB was elevated [99, 100]. Additional experiments showed that lncRNA-ATB adsorbed miR-200c and released ZEB1, promoting the occurrence of EMT [99, 100].

Yan et al. [94, 95] detected that the expression level of miR-503 was decreased in a murine model of silicosis. Cell experiments showed that the expression of lncRNA-MALAT1 was increased in HBE and A549 cells treated with silica dust, and miR-503 was decreased. The researchers also demonstrated that lncRNA-MALAT1 could promote EMT by regulating the PI3K/AKT/SNAIL-signaling pathway by targeting miR-503 and targeting PI3K and p85 through the dual-luciferase reporter assay system, lncRNA overexpression, and siRNA interference [94, 95].

*The Association of Different Cells.* Wu et al. [92, 93] ascertained that lncRNA-CHRF expression was increased, and miR-489 expression was decreased in a murine model of silicosis, in SiO_2_ dust–treated macrophages (mouse RAW264.7 and human THP-1 macrophages), and in NIH/3T3 cells and MRC-5 cells treated with TGF-*β*. miR-489 inhibited the expression of MyD88, IL-1*β*, and TGF-*β* in mice and macrophages [92, 93]. miR-489 inhibited total SMAD3 and phosphorylated SMAD3 (p-SMAD3) levels and alleviated pulmonary fibrosis in mice and fibroblasts [92, 93]. They also proved that lncRNA-CHRF could adsorb miR-489 and promote fibrosis [92, 93].

Lei et al. [137] mined the microarray data in the GEO and discovered 1,140 differentially expressed mRNAs and 1,406 differentially expressed lncRNAs after BEAS-2B cells were exposed to unshaped nanosilica particles; among these findings, 20 differentially expressed mRNAs were upregulated, 1,120 differentially expressed mRNAs were downregulated, 213 differentially expressed lncRNAs were upregulated, and 1,193 differentially expressed lncRNAs were downregulated. The study also demonstrated through cell experiments that Ak131029 inhibition can inhibit the proliferation of human lung fibroblasts (HPFS and HS888LU) [137].

Xu et al. [91] found that lncRNA-HOTAIR promoted inflammation by adsorbing miR-326. The expression of miR-326 was decreased in the lung tissue of this murine model of silicosis [91]. Tumor necrosis factor superfamily 14 (TNFSF14) and polypyrimidine bundle binding protein 1 (PTBP1) were identified as targets of miR-326 [91]. *In vitro* experiments showed that miR-326 inhibited pulmonary inflammation by targeting TNFSF14 in silica-treated lung epithelial cells (HBE and A549) and promoted autophagy activity by targeting PTBP1 in TGF-*β*–stimulated MRC-5 cells and NIH/3T3 cell models [91].

Because the study of lncRNA started later than that of miRNA, the pathogenic mechanism of lncRNA in pneumoconiosis has not been as thoroughly studied. The application of lncRNA in the treatment of pulmonary fibrosis, cardiac fibrosis, renal fibrosis, and liver fibrosis remains in the exploratory stage and has not been approved clinically. The treatment of lncRNA in pneumoconiosis can be explored by referring to the research on therapeutic targets of other diseases.

## 4. Crosstalk among Different Epigenetic Changes

The occurrence and development of pneumoconiosis involve the changes of multiple genes, and the regulation of gene expression involves many steps. Studies often combine several different mechanisms in series. As mentioned earlier, miRNA expression is regulated by epigenetics, such as DNA methylation, lncRNA, circRNA, and histone modification [53–59, 138], and lncRNA expression is also regulated by transcription factors or epigenetics [113–115]. miRNAs can indirectly regulate gene expression by regulating HDAC [65] and DNA methylase [66]. In addition, miRISC promotes mRNA degradation and directly inhibits mRNA translation into protein [61]. Some pre-miRNAs in the nucleus can also competitively bind repressor proteins and facilitate mRNA translation into proteins [67, 68]. lncRNAs located in the cytoplasm can promote the translation of mRNA through the mechanism of ceRNAs [91–95, 99, 100], or inhibit the translation of mRNA [126], or directly promote the degradation of mRNA [127, 128]. The ceRNA mechanism of lncRNA-miRNA in pneumoconiosis will not be repeated here, but crosstalk among several other different epigenetic changes, including several other epigenetic cascades, will be described below.

Yang et al. [55, 56] detected that zinc finger CCCH-type containing 4 (ZC3H4) increased in lung tissue of silicosis mice and in RAW264.7 cells stimulated by silica. Cell experiments *in vitro* showed that macrophages are activated and that circular ZC3H4 (circZC3H4) RNA increased after silica stimulation [55, 56]. CircZC3H4 binds and inhibits miR-212, promotes the expression of ZC3H4, and causes the migration of the murine fibroblast cell line L929 [55, 56]. In addition, Jiang et al. [54] ascertained the upregulation of ZC3H4 in alveolar epithelial cells in lung tissue samples of patients with silicosis and mice with silicosis. Through *in vitro* experiments of the murine lung epithelial cell line MLE-12, knockdown of ZC3H4 prevented EMT and migration caused by SiO_2_ [54]. CircZC3H4 adsorbed miR-212 and regulated the expression of ZC3H4 [54].

## 5. Conclusion

Pulmonary fibrosis is a typical pathological change of pneumoconiosis. The formation of fibrosis is the basic response of the body to resist pathogens and normal wound healing [139]. In pulmonary fibrosis, disease-specific triggers cause inflammation, myofibroblast activation, and activation of a profibrotic positive feedback loop, resulting in the persistence and amplification of fibrosis [140, 141]. Although the triggers, susceptibility, and initial inflammatory responses of various pulmonary fibrosis diseases are different, the current hypothesis is that they share common regulatory mechanisms, such as epigenetic regulation, at later stages. At present, miRNA is the most studied epigenetic field in pneumoconiosis. Studies mainly focus on actions of miRNA and fibrosis-related mRNA to form miRISC, which in turn affects fibrosis. Although many epigenetic regulation mechanisms of lncRNA exist, research in pneumoconiosis is mainly focused on the formation of ceRNA with miRNA. The study of DNA methylation in pneumoconiosis is still in an early, observational stage. Antifibrosis effects of epigenetic factors in pneumoconiosis have mainly been explored in cell and animal experiments, and the therapeutic potential of epigenetic factors remains to be explored. Some clinical trials have explored epigenetics for the treatment of renal fibrosis, but few clinical studies have focused on the treatment of pulmonary fibrosis [52, 111].

Exploration of epigenetic changes in pneumoconiosis is of great value for understanding the pathogenesis and progression of pneumoconiosis, disease surveillance, diagnosis, intervention, and treatment. For example, the development of epigenetic biomarkers with diagnostic value in urine or blood is ideal because it can exempt subjects or patients from the usual invasive tissue sample collection. In addition, epigenetic modulators open up new ideas for the treatment of pneumoconiosis. Clinical data have shown that new epigenetic drugs have good therapeutic potential in human cancers. The first generation of epigenetic modulators approved for clinical treatment includes nonselective DNMT and HDAC inhibitors for myelodysplastic syndrome and cutaneous T cell lymphoma [142, 143]. lncRNA-PCA3 is the first and only ncRNA approved by the Food and Drug Administration as a cancer biomarker detection so far [144]. All of these provide some key experiences and lessons for pneumoconiosis. Searches for new targets using epigenetic factors could identify new ideas for the treatment of pneumoconiosis and even pulmonary fibrosis. The vast promise of clinical research on epigenetic drugs may enable the full potential of these therapies to be revealed in the near future.

## Figures and Tables

**Table 1:** Main miRNA changes involved in pneumoconiosis.

miRNA	Regulation	Model						Target gene and signal pathway	Reference
		Patients	*In vivo*		*In vitro*				
			Rat	Mouse	Macrophage	Fibroblast	Alveolar epithelial cell		
miR-21/21-5p	Up	√	√	√					[69–72]
miR-96/96-5p	Up	√	√						[68, 73–75]
miR-107-3p	Up	√			RAW264.7			miR-107-3p can inhibit the expression of chordin and its downstream BMP-2, SMAD1/5/9, and ID-1, which is related to the activation of TGF-*β* signaling pathway.	[76–78]
miR-122-5p	Up	√			RAW264.7				[74–78]
miR-125a-5p	Up	√			RAW264.7	IMR-90		(1) miR-125a directly inhibited the expression of hnRNP K and EZH2 (2) miR-125a-5p can promote the proliferation and apoptosis of NIH/3T3 and promote the occurrence of fibrosis	[68, 74, 75]
miR-132-3p/132-5p/212-3p/212-5p	Up		√						[71]
miR-155/155-3p	Up	√	√						[69, 71]
miR-291a-3p/291b	Up		√						[71]
miR-292-3p/292-5p	Up		√						[71]
miR-293-5p	Up		√			Rat primary lung fibroblasts			[71, 79]
miR-320a/320b/320c	Up	√							[80]
miR-335-5p	Up	√			RAW264.7				[76–78]
miR-466b-2-3p/466b-3p/466b-4-3p/466b-5p	Up		√						[71]
miR-743a-3p/743b-3p/743b-5p	Up		√						[71]
miR-26a/26a-5p/26b	Down		√						[72, 73]
miR-27b-5p	Down	√			RAW264.7				[76–78]
miR-34a-5p/34c-3p/449a	Down	√	√	√		NIH/3T3, MRC-5	A549	miR-34a-5p may play a potential role in inhibiting EMT by inhibiting SMAD4.	[72, 81–84]
miR-99a/99a-5p	Down	√	√						[73, 80]
miR-101/101a	Down		√		NR8383			Overexpressed miRNA-101 in BMSCs can inhibit ATG4D by regulating the endoplasmic reticulum stress of NR8383 cells, and effectively inhibit the autophagy of macrophages caused by silica.	[73, 85–87]
miR-140/140-5p	Down	√	√						[73, 80]
miR-149	Down	√		√			A549, HBE	Overexpressed miR-149 can inhibit the expression of IL-6 *in vitro*.	[88, 89]
miR-151a-3p	Down	√							[68, 74, 75]
miR-181b	Down		√		NR8383				[73]
miR-222	Down	√							[88, 90]
miR-326	Down			√			A549, HBE	(1) miR-326 inhibited pulmonary inflammation by targeting TNFSF14 in silica-treated lung epithelial cells (2) miR-326 promoted autophagy activity by targeting PTBP1 in TGF-*β*–stimulated MRC-5 cells and NIH/3T3 cells models	[91]
miR-370-3p	Down		√			Rat primary lung fibroblasts			[71, 79]
miR-379-3p/379-5p	Down		√						[71]
miR-409-3p/409a-5p	Down	√	√						[68, 71, 73–75]
miR-411-3p/411-5p	Down		√			Rat primary lung fibroblasts		(1) miR-411-3p effectively reduced the progression of silicosis or fibroblast fibrosis by regulating the expression of MRTFA *in vivo* and *in vitro* (2) miR-411-3p could inhibit the expression of SMURF2 and reduce the ubiquitination degradation of SMAD7 regulated by SMURF2, thereby blocking the TGF-*β*/SMAD signaling pathway and alleviating silicosis pulmonary fibrosis by stimulating the primary lung fibroblasts of rats with TGF-*β*	[71, 79]
miR-486-5p	Down	√		√		NIH/3T3		miR-486-5p could bind to SMAD2 and participate in the process of pulmonary fibrosis through TGF-*β* signaling pathway.	[70]
miR-489	Down			√	RAW264.7, THP-1 macrophages	NIH/3T3, MRC-5		(1) miR-489 inhibited the expression of MyD88, IL-1*β*, and TGF-*β* in mice and macrophages (2) miR-489 inhibited total SMAD3 and p-SMAD3 levels and alleviated pulmonary fibrosis in mice and fibroblasts	[92, 93]
miR-503	Down			√			A549, HBE	miR-503 was adsorbed by lncRNA-MALAT1 and downregulated miR-530, increased PI3K and p85, and promoted EMT by regulating the PI3K/AKT/SNAIL signaling pathway.	[94, 95]
miR-542-5p	Down			√		NIH-3T3, MRC-5		Overexpressed miR-542-5p inhibited ITGA6, which in turn inhibited the phosphorylation of FAK/PI3K/AKT and alleviated fibrosis.	[96]
miR-1193-3p	Down		√			Rat primary lung fibroblasts			[71, 79]
Let-7a-3p/let-7a-5p/let-7d/let-7d-3p/let-7d-5p/let-7f/let-7i-5p	Down	√	√			IMR-90	A549	(1) Let-7a-5p has four target genes: BCL2L1, FAS, MAPK8, and IGF1R (2) The downregulated let-7d in A549 cells can increase the expression of HMGA2 and promote EMT	[68, 73–75, 97]
miR-16/16-2-3p/16-5p	Discord	√							[69, 80]
miR-22/22-5p	Discord	√	√						[73, 80]
miR-25/25-3p	Discord	√	√						[73, 80, 88]
miR-29a/29b/29b-3p/29c	Discord	√	√				A549, RLE-6TN	miR-29b attenuated or even reversed the EMT process induced by silica *in vivo* and *in vitro* experiments.	[69, 72, 73, 89, 98]
miR-30a/30c/30c-2-3p/30d/30e	Discord	√	√		RAW264.7				[73, 76–78]
miR-126/126a-5p	Discord	√	√		RAW264.7				[73, 76–78]
miR-146a/146b/146b-3p/146b-5p	Discord	√	√		NR8383				[68, 71, 73–75]
miR-200a-5p/200b/200b-3p/200c/200c-3p	Discord	√	√				A549, Beas-2B	miR-200c was adsorbed by lncRNA-ATB to release ZEB1 and promote the occurrence of EMT.	[89, 98–101]
miR-204	Discord	√	√						[69, 73]
miR-300-3p/300-5p	Discord		√						[71, 73]
miR-423-3p/423-5p	Discord	√	√						[68, 72, 74, 75]

**Table 2:** The main lncRNA changes involved in pneumoconiosis.

lncRNA	Regulation	Model						Target gene and signal pathway	Reference
		Patients	*In vivo*		*In vitro*				
			Rat	Mouse	Macrophage	Fibroblast	Alveolar epithelial cell		
lncRNA-ATB	Up	√					A549, Beas-2B	lncRNA-ATB adsorbed miR-200c and released ZEB1, promoting the occurrence of EMT.	[99–101]
lncRNA-MALAT1	Up						A549, HBE	lncRNA-MALAT1 can promote EMT by regulating the PI3K/AKT/SNAIL-signaling pathway by targeting miR-503 and targeting PI3K and p85.	[94]、 [95]
lncRNA-CHRF	Up				RAW264.7, THP-1 macrophages	NIH/3T3, MRC-5		lncRNA-CHRF can adsorb miR-489. (1) miR-489 inhibited the expression of MyD88, IL-1*β* and TGF-*β*1 in mice and macrophages (2) miR-489 inhibited total SMAD3 and p-SMAD3 levels and alleviated pulmonary fibrosis in mice and fibroblasts	[92]、 [93]
lncRNA-HOTAIR	Up						A549, HBE	lncRNA-HOTAIR regulates the pulmonary inflammatory process by targeting miR-326 to regulate TNFSF14.	[91]
LOC103691771	Up		√			Rat primary lung fibroblasts		LOC103691771 promotes differentiation of myofibroblasts by regulating the TGF-*β*–SMAD2/3 signaling pathway.	[134]
LOC102549714	Up		√						[134]
LOC102550137	Up		√						[134]
NONRATT015512.2	Up		√						[135]
NONRATT017365.2	Up		√						[135]
ENSRNOT00000054583.1	Up		√						[135]
ENSRNOT00000048692	Up		√						[135]
ENSRNOT00000054495.1	Up		√						[135]
NONRATT016740.2	Up		√						[135]
NONRATT006104.2	Up		√						[135]
NONRATT002987.2	Up		√						[135]
NONRATT009275.2	Up		√						[135]
ENSRNOT00000001712	Up		√						[135]
LOC103693125	Down		√						[134]
LOC103692016	Down		√						[134]
NONRATT030230.2	Down		√						[135]
NONRATT024771.2	Down		√						[135]
NONRATT009769.2	Down		√						[135]
NONRATT022710.2	Down		√						[135]
NONRATT001412.2	Down		√						[135]
NONRATT011944.2	Down		√						[135]
NONRATT011539.2	Down		√						[135]
ENSRNOT00000014917	Down		√						[135]
ENSRNOT00000083823.1	Down		√						[135]
NONRATT029573.2	Down		√						[135]

## Data Availability

All data, models, and code used during the study appear in the submitted article.
